# Assessment of Intestinal Immunity and Permeability of Broilers on Partial Replacement Diets of Two-Stage Fermented Soybean Meal by *Bacillus velezensis* and *Lactobacillus brevis ATCC 367*

**DOI:** 10.3390/ani11082336

**Published:** 2021-08-08

**Authors:** Chia Fen Tsai, Li Jen Lin, Chao Humg Wang, Ching Sung Tsai, Shang Chang Chang, Tzu Tai Lee

**Affiliations:** 1 Department of Animal Science, National Chung Hsing University, Taichung 402, Taiwan; bbella1123tw@gmail.com; 2 School of Chinese Medicine, College of Chinese Medicine, China Medical University, Taichung 404, Taiwan; linlijen@mail.cmu.edu.tw; 3 Central Union Oil Corporation, Taichung 436, Taiwan; wjh.0910@cuoc.com.tw (C.H.W.); tcs.0929@cuoc.com.tw (C.S.T.); 4 Kaohsiung Animal Propagation Station, Livestock Research Institute, Council of Agriculture, Kaohsiung 912, Taiwan; macawh@tlri.gov.tw; 5 The iEGG and Animal Biotechnology Center, National Chung Hsing University, Taichung 402, Taiwan

**Keywords:** soybean meal, soy peptide, two-stage fermentation, immunity, intestinal morphology, tight junction, broiler

## Abstract

**Simple Summary:**

Inflammation induced by diet, environmental factors, and stimulated innate immunity is not conducive to intestinal maintenance and remodeling. Fermented soybean meal (FSBM) by *Bacillus velezensis* (Bv) and *Lactobacillus brevis ATCC 367* (Lb) reduces these negative factors and provides bioactive active peptides that are beneficial to intestinal repair and regulate the immune system of the intestinal tract. This study showed that two-stage FSBM regulates the immunity and tight junction in the jejunum, which are beneficial to health and performance.

**Abstract:**

The effect of soybean peptides from fermented soybean meal on the intestinal immunity and tight junction of broilers was assessed. Roughly, two-stage fermented soybean meal prepared with Bv and Lb (FSBM_B+L_), which has nearly three times higher soluble peptides than soybean meal (SBM), and reduced galacto-oligosaccharide (GOS) content and allergen protein. The one-stage fermented by Bv (FSBM_B_) has the highest soluble peptides, while commercial lactic acid bacteria (FSBM_L_) has the highest Lactic acid bacteria count; these were used to compare the differences in the process. Ross308 broilers (n = 320) were divided into four groups: SBM diet and a diet replaced with 6% FSBM_B+L_, FSBM_B_, or FSBM_L_. The growth performance was recorded during the experiment, and six birds (35-day-old) per group were euthanized. Analysis of their jejunum and ileum showed that the fermented soybean meal significantly improved the villus height in the jejunum (*p* < 0.05) and reduced the crypt hyperplasia. The FSBM_B_ group had the highest reducing crypt depth; however, the FSBM_B+L_ group had the highest villus height/crypt depth in the ileum (*p* < 0.05). In the jejunum, the relative mRNA of *CLDN-1* and *Occludin* increased 2-fold in the treatments, and *ZO-1* mRNA increased 1.5 times in FSBM_L_ and FSBM_B+L_ (*p* < 0.05). Furthermore, the level of *NF-κB* and *IL-6* mRNAs in FSBM_L_ increased, respectively, by 4 and 2.5 times. While FSBM_B_, along with FSBM_B+L_, had a 1.5-fold increase in the mRNA of *IL-10*, that of *NF-κB* increased 2-fold. FSBM_B+L_ and FSBM_B_ singly led to a 2- and 3-fold increase in *IL-6* mRNA, respectively (*p* < 0.05). FSBM_B_ and FSBM_B+L_ can also upregulate *MUC2* in the jejunum (*p* < 0.05). In short, using the soybean peptides from two-stage fermented soybean meal can ameliorate the negative factors of SBM and effectively regulate immune expression and intestinal repair, which will help broilers maintain intestinal integrity.

## 1. Introduction

Soybean meal (SBM) has 44 to 48% crude protein content [1]. It is a suitable material for the production of soy peptide, which exhibits potent bioactivity including antioxidant, anti-inflammatory, and ACE-inhibitory activity and has antimicrobial and anticancer properties. On the other hand, the fermented soybean meal (FSBM) used in animals’ diets also enhances growth performance [2], redox status [3,4], and immune regulation [5]. However, the main bioactive ingredient in FSBM that affects livestock after intake needs to be examined.

The influence of FSBM on the host are divided into two categories: (I) Reduction in the antigen from glycinin (11S globulin, 40% of total protein) and β-conglycinin (7S globulin, 30% of total protein) after enzyme hydrolysis leads to better absorption of FSBM, down-regulation of the *NF-κB* pro-inflammatory pathway [6], and then restoration of the intestinal tight junction proteins [7]; (II) soy isoflavone release and deglycosylase after the hydrolysis of cellulose, hemicellulose modified by β-glucosidase [8], and soy peptides produced by the hydrolysis of glycinin and β-conglycinin [9]. While these compounds provide FSBMs bioactivity, soy peptides are considered to have more potential for FSBM development.

According to Sanjukta [10] and Hou [11], hydrolysis of bioactive soy peptides from 65 to 85% of the structural protein produce 2 to 20 amino acid residues. From the digestive fluid, these peptides pass through the duodenum and act to scavenge hydroxyl radicals and chelate transition-metal ions [12]. The structure or amino acid composition peptides also help in regulating pro-inflammatory factors in the intestinal epithelium, including Th1 (interleukin, IL-12, TNF-α, and IFN-γ), Th17 (TGF-β, IL-6, and IL-17) related cytokines, and IL-10 at the regulatory T cell [6]. When peptides arrive at the jejunum, they can be transferred by peptide transporter 1 (PepT1), or be passively transported through tight junction protein, or through pinocytosis [13]. The bioactive peptides can be transported through serum and continue activating the target organ (liver, spleen, and breast).

So far, the functions of soy peptides have been confirmed in vivo animal experiments, including mice [14] and piglet [15], but there have been only a few studies on poultry, especially broilers. For nearly half a century, dealing with commercial demand, industrial production of broilers has been accelerated. This has worsened the stress in birds, including oxidative stress and potential pathogen and environmental stress [16]. The gastrointestinal tract, especially complex microbiota of feces, feed material, and foreign factors, are consider to be vital against oxidative stress and potential pathogens [17]. Soy peptides are usually present in FSBM in livestock diets [11]; however, only a few studies have discussed the effect of soy peptides in two-stage FSBM on the animals’ intestinal immunity and permeability. In recent studies, a partial replacement diet from 3% to 6% showed better growth performance, immunity, and intestinal morphology [4,5]. Cheng’s group, using two-stage fermentation, showed reducing anti-nutrition factors and increasing with <6 kDa soy peptide content, which decreased the serum IgG and downregulated spleen *IL-4* and *IL-10* mRNA levels by partial replacement in 10% of broiler diets [18]. However, more investigation in needed regarding the relationship between the FSBM content and the intestinal cell repair ability, which is associated with immune response in broilers. We hypothesized that FSBM can regulate the immune response and restore epithelial cell repair ability due to glycinin hydrolysis. This article focuses on the effects and associated molecular mechanisms of the soy peptides included in FSBM on broilers’ intestinal immunity and permeability. 

## 2. Materials and Methods

### 2.1. Preparation and Characteristics of FSBM

Lb and Bv were used for SBM fermentation. First, enrichment of Lb was carried out in de Man, Rogosa, and Sharpe (MRS) broth at 30 °C under anaerobic conditions for 48 h; enrichment of Bv was done in Luria–Bertani (LB) broth at 37 °C in aerobic conditions for 24 h. Fifty grams of commercial SBM from Central Union Oil Corporation (Taichung, Taiwan) was sterilized at 121 °C for 15 min. Initial moisture was adjusted to 50% after cooling the SBM and fermented under the following conditions: one-stage fermentation after inoculating Bv 2.5% in aerobic fermentation for 60 h (FSBM_B_); two-stage fermentation after inoculating Bv 2% in aerobic fermentation for 24 h, and then inoculating Lb in anaerobic fermentation for 36 h (FSBM_B+L_). After fermentation, 1 g fresh fermented product was collected for live cell count. The remaining portions were dried at 55 °C for 12 h and ground for composition analysis according to AOAC [19], including dry matter (DM) and crude protein (CP). Anti-nutrition factors were measured using a commercial ELISA kit for allergen protein (Biofront, Tallahassee, FL, USA), trypsin inhibitor (Eurofins Immunolab, Kassel, Germany), and high-performance liquid chromatography (HPLC) for GOS according to Faridah [20] and Yin’s [21] method using a column (ZORBAX carbohydrate, 4.6 mm × 150 mm, 5 μm). The TCA-soluble protein content was as per the method by Xie [22]. All of FSBM’s composition were showed as Table 1. Then, 20 kg FBSM was processed for the animal trials after measuring the contents.

### 2.2. Animal Experiment

#### 2.2.1. Animal Feeding and Housing

The feeding trial was conducted during summer at the National Chung Hsing University (NCHU) Experimental Husbandry Farm (Taichung, Taiwan) with an average environmental temperature of 30 ± 2 °C and average environmental humidity of 77 ± 11%. The protocols for feeding and housing were carried out according to the Animal Care and Use Committee, NCHU (IACUC: 109-055). Three hundred and twenty Ross 308 broilers, one-day-old (initial weight 44 ± 1.2 g) were categorized into four groups with four replicates, with feeding and water drinking *ad libitum*. The groups were corn-soybean meal (SBM) and diets replaced by 6% FSBM_B_, FSBM_B+L_, and commercial FSBM fermented by *Lactobacillus spp*. (FSBM_L_). The feeding formula for the starter (day 1–21) and finisher (day 22–35) was as per the NRC (1994), with equal amounts of protein and energy showed as Table 2. Per pen from day 1, the temperature (34 ± 1 °C) was slowly downgraded to room temperature by day 7 (27 ± 1 °C) and kept as such until the experiment ended.

#### 2.2.2. Growth Performance and Sample Collection

When the birds were 21 and 35 days old, the body weight (BW) and feed intake (FI) for each group with replicate were measured, then the body weight gain (BWG) and feed conversion rate (FCR) were calculated. On day 35, serum samples from the wing vein of eight birds from each group were collected intravitally. Serum samples were kept at 4 °C for 4 h and centrifuged at 3000 rpm at 4 °C for 10 min. Six birds within the average weight from each group were then selected for euthanizing and sampling. For intestinal morphology and RT-qPCR analysis, birds were fasted for 24 h and euthanized by cervical dislocation, then 2 cm of the middle section of the jejunum and ileum were sampled and rinsed by phosphate buffer solution. Samples were soaked in 10% formalin solution at room temperature for intestinal morphology and RNA shield (Zymo research CO., Irvine, CA. USA) treatment at −20 °C for RNA extraction. For quantitative estimation of ILs, the samples (jejunum, serum) were stored at −80 °C for future use by a commercial ELISA kit (FineTest, Wuhan, China).

The samples for intestinal morphology were embedded by paraffin and stained with hematoxylin and eosin. The slices was observed under a light microscope using the Mosaic 2.1 analysis system (Tucsen Photonics Co., Ltd., Fujian, China). For each treatment, 30 images were acquired for the measurement and calculation of villus height and crypt depth.

#### 2.2.3. Jejunum Total RNA Isolation and qPCR

The sample (0.1 g) was taken from the RNA shield, soaked in RNAzol (Molecular research center, Ohio, USA), and macerated in a lysis tube (Zymo research CO., Irvine, CA, USA). The supernatant was extracted following the protocol in the commercial kit (Zymo research CO., Irvine, CA, USA). A Prime Script™ RT reagent Kit with gDNA Eraser (Applied Biosystems, Waltham, MA, USA) was used for reverse transcription of extracted RNA. The qRT-PCR analysis was conducted on the StepOnePlus™ Real-Time PCR System (Thermo Fisher, Waltham, MA, USA). The dilution of cDNA and primer was carried out according to the included protocol. The PCR mix consisted of 1.2 μL cDNA samples, 5 μL 2× SYBR GREEN PCR Master Mix-ROX (Appliedbiosystems, Waltham, MA, USA), 1.8 μL deionized water, and 1 μL forward and reverse primers. The performance of qRT-PCR was used to measure relative mRNA expression level by the 2^−^^△△^^Ct^ method. While *β-actin* was used for the housekeeping gene, the genes of *Gallus gallus* (chicken) were used as gene-specific primers, as listed in Table 3.

### 2.3. Statistical Analysis

The experimentation of the fermented products was carried out in triplicate. SAS software (SAS^®^ 9.4, 2016, SAS Institute Inc., Cary, NC, USA) with analysis of variance mode was used to analyze all data variance. The differences between treatment means were separated using Duncan’s multiple range test with *p*-value < 0.05.

## 3. Results

### 3.1. The Composition and Enzyme Activity of SBM and FSBM by Bv and Lb

**Figure 1:**
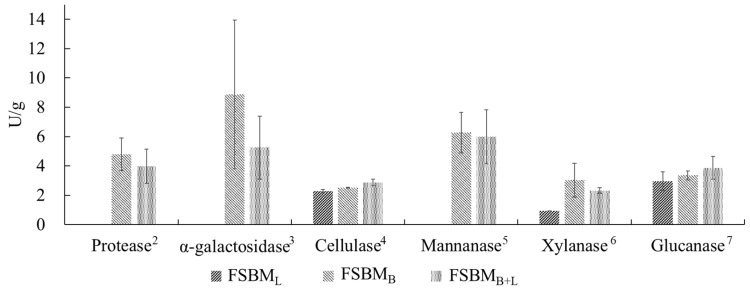
Enzymes activities of FSBM products. Each value represents the mean ± standard deviation (n = 3). ^2^ One unit was defined as the hydrolysis of 1 mg mL^−^^1^ azocasein to change the absorbance by 0.001. ^3^ One unit was defined as 2 mM p-Nitrophenol α-D-Galactopyranoside hydrolysis to release 1 μM p-nitrophenol. ^4^ One unit was defined as the consumption of 5 mg mL^−^^1^ CMC to generate 1 μM of reducing sugar. ^5^ One unit was defined as the consumption of 3 mg mL^−^^1^ locust bean gum to generate 1 μM of D-mannose. ^6^ One unit was defined as the consumption of 10 mg mL^−^^1^ beechwood xylan to generate 1 μM of D-xylose. ^7^ One unit was defined as the consumption of 4 mg mL^−^^1^ β-glucan to generate 1 μM of reducing sugar. SBM: Soybean meal; FSBM_L_: commercial control; FSBM_B_: SBM one-stage fermented by Bv; FSBM_B+L_: SBM two-stage fermented by Bv and Lb.

Table 1 presents the changes in the composition of the feeds after two-stage fermentation. Compared with SBM, the TCA-soluble protein of the FSBM_L_ increased by 176%, that of FSBM_B+L_ increased by 244%, and that of FSBM_B_ increased by 360% (*p* < 0.05). Concurrently, the contents of stachyose and raffinose in the three treatments reduced significantly (*p* < 0.05). Among the treatments, the stachyose content of FSBM_B_ and FSBM_B+L_ were below the detection limit (0.234 mg mL^−^^1^). Allergic protein and trypsin inhibitor decreased significantly in three treatments, the lowest content being in FSBM_B_ (*p* < 0.05). After adjusting the pH value to 5.5 for enzyme activity assay, protease and α-galactosidase activity was not detected in FSBM_L_, while FSBM_B_ had the highest protease, α-galactosidase, mannanase, and xylanase activities of about 4.79, 8.87, 6.27, and 3.03, respectively. The results of other enzyme activities of the fermented products are shown in Figure 1. 

### 3.2. Effect of Two-Stage FSBM on Growth Performance of 1 to 35-Day-Old Broilers

**Figure 2:**
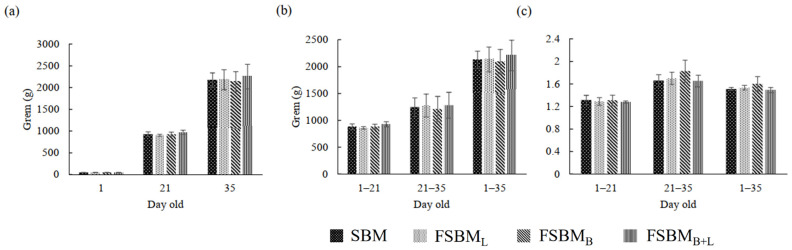
Effect of two-stage FSBM on Body weight (**a**), body weight gain (**b**), and feed conversion ratio (**c**) of broilers. Each value represents the mean ± standard deviation (n = 4). SBM: Soybean meal; FSBM_L_: commercial control; FSBM_B_: SBM one-stage fermented by Bv; FSBM_B+L_: SBM two-stage fermented by Bv and Lb.

Figure 2a–c reveals that, among the four groups, there was no significant difference in BW, BWG, and FCR (*p* > 0.05). When birds were 35 day-old, the average weights of the four groups were 2171 g, 2177 g, 2136 g, and 2252 g, respectively.

### 3.3. Effect of Two-Stage FSBM on Intestinal Morphology of 35-Day-Old Broilers

**Figure 3:**
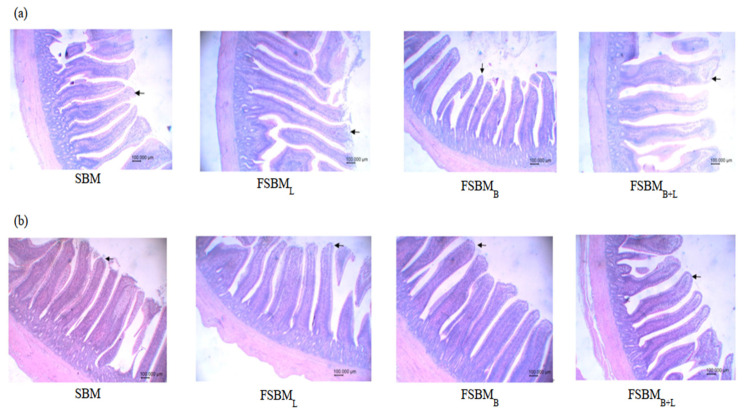
Effect of two-stage FSBM on Jejunum (**a**) and ileum (**b**) intestinal morphology of 35 day-old broilers. SBM: Soybean meal; FSBM_L_: commercial control; FSBM_B_: SBM one-stage fermented by Bv; FSBM_B+L_: SBM two-stage fermented by Bv and Lb.

In the jejunum (Figure 3a), we observed that SBM had a thin villus with a thickened, proliferated crypt compared to the other groups. FSBM_B+L_ had a less proliferated crypt. In the ileum (Figure 3b), four groups had similar villus apparent traits, but SBM had the same status as the jejunum on the proliferated crypt. According to the images we captured, the intestinal morphology results are shown in Table 4. All three treatments significantly increased the villus height; only FSBM_B_ significantly decreased the crypt depth compared with the other groups (*p* < 0.05). In the ileum, FSBM_B+L_ had a higher villus height/crypt depth ratio (*p* < 0.05).

### 3.4. Effect of Two-Stage FSBM on Serum and Jejunum’ s Immunity of 35 Day-Old Broilers

The quantitative pro-inflammatory cytokine detected in the serum and jejunum of broilers is listed in Table 5. FSBM_L_ significantly increased IL-1β, IL-10, and IL-6 in the jejunum, compared with those in the other groups (*p* < 0.05). FSBM_L_, FSBM_B_, and FSBM_B+L_ had significantly decreased serum IL-1b at 47, 20, and 39 pg mL^−1^, respectively (*p* < 0.05).

### 3.5. Effect of Two-Stage FSBM on Relative mRNA Expression in Jejunum of 35 Day-Old Broilers

**Figure 4:**
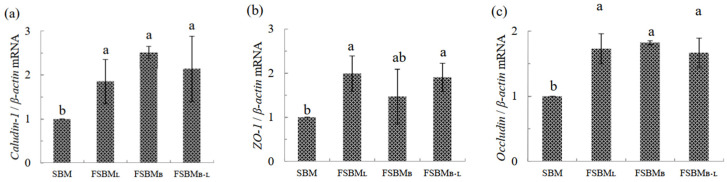
Effect of two-stage FSBM on jejunum’ s relative mRNA expression of genes related to *Caludin-1* (**a**), *ZO-1* (**b**), and *Occludin* (**c**) of 35 day-old broilers. Each value represents the mean ± standard deviation (n = 5). ^a, b^ Means within a row with different letters differed significantly (*p <* 0.05). SBM: Soybean meal; FSBM_L_: commercial control; FSBM_B_: SBM one-stage fermented by Bv; FSBM_B+L_: SBM two-stage fermented by Bv and Lb.

The relative mRNA expression of tight junction protein is shown in Figure 4a–c. Compared with SBM, Caludin-1 in FSBM_L_, FSBM_B_, and FSBM_B +L_ was significantly upregulated by about 1.9, 2.5, and 2.1 times, respectively (*p* < 0.05). In both FSBM_L_ and FSBM_B+L_, ZO-1 was significantly upregulated by about 2 times (*p* < 0.05), while it was upregulated 1.5 times in FSBM_B_, but there was no significant difference compared to SBM (*p* > 0.05). All three treatments significantly upregulated Occuldin by about 1.7 times, but there was no significant difference between each group.

**Figure 5:**
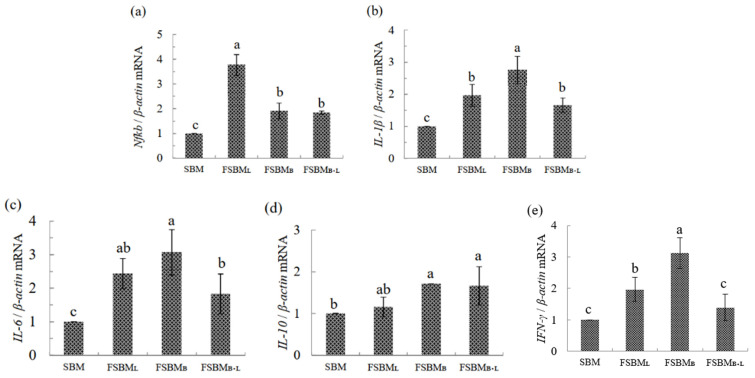
Effect of two-stage FSBM on jejunum’ s relative mRNA expression of genes related to *NF-κB* (**a**), *IL-1β* (**b**), *IL-6* (**c**), *IL-10* (**d**), and *IFN-γ* (**e**) of 35 day-old broilers. Each value represents the mean ± standard deviation (n = 5). ^a–c^ Means within a row with different letters differed significantly (*p <* 0.05). SBM: Soybean meal; FSBM_L_: commercial control; FSBM_B_: SBM one-stage fermented by Bv; FSBM_B+L_: SBM two-stage fermented by Bv and Lb.

The relative mRNA expression of the pro-inflammatory cytokines is shown in Figure 5a–e. Compared to SBM, FSBM_B_ had significantly upregulated NF-κB, by about 2 times the amount, and that in FSBM_L_ was upregulated by about 4 times the amount (*p* < 0.05). IL-1β in FSBM_B_ was significantly upregulated by about 2.5 times, while that in FSBM_L_ and FSBM_B+L_ was significantly upregulated by about 2 times (*p* < 0.05). The IL-6 in FSBM_B+L_ was upregulated by about 2 times the amount, while that in FSBM_L_ and FSBM_B_ was upregulated by about 2.5 times and 3 times, respectively (*p* < 0.05). IL-10 was only significantly upregulated by 1.5 times the amount compared to SBM (*p* < 0.05), while IFN-γ was significantly upregulated by 3 times in FSBM_B_ compared to SBM.

**Figure 6:**
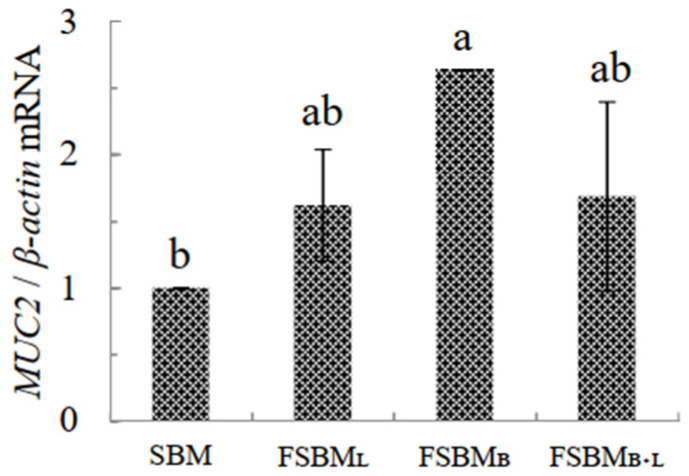
Effect of two-stage FSBM on jejunum’ s relative mRNA expression of genes related to *MUC2* of 35 day-old broilers. Each value represents the mean ± standard deviation (n = 5). ^a, b^ Means within a row with different letters differed significantly (*p <* 0.05). SBM: Soybean meal; FSBM_L_: commercial control; FSBM_B_: SBM one-stage fermented by Bv; FSBM_B+L_: SBM two-stage fermented by Bv and Lb.

The relative mRNA expression of the MUC2 is shown in Figure 6. The level of MUC2 in FSBM_B_ was significantly upregulated 2.6 times more than that in other treatments compared to SBM (*p* < 0.05).

### 3.6. Effect of Two-Stage FSBM on Economic Benefits

An evaluation of the economic benefits of adding EP to the broiler diet is summarized in Table 6. The income over feed cost (IOFC) of the control, FSBM_L_, FSBM_B_, and FSBM_B+L_ groups were 61.9, 59.9, 57.2, and 63.3 TWD/bird, respectively.

## 4. Discussion

Past studies have suggested that partial replacement of dietary SBM by FSBM improved the broiler’s BWG and FCR due to increased protein digestibility [23]. Although fermentation with the soy peptide upgraded the content, only a 3 to 6% partial replacement diet could reach a similar effect [5,24]. FSBM_B+L_ showed a better FCR through the entire feeding period compared to SBM, and we observed that FSBM_B+L_ increased the income over feed cost, which means FSBM_B+L_ has the potential to bring more economic value. This may be due to the fact that the nutrients and peptides in FSBM_B+L_ were better absorbed and utilized by the chickens, at the same time ensuring the birds’ increased protein intake, which corresponds to the effects of fermented products on animal nutrition seen previously.

The GOS from SBM could not be digested by the digestive enzymes of monogastric animals. In young monogastric animals, it may cause nutritional diarrhea and lead to serious gut injury at the distal end of the intestine [25,26]. The jejunum is the main absorption part of the digestive tract. If gut epithelial cells cannot renew in time, the crypt shows compensatory hyperplasia [6]. From our results, FSBM had reduced hyperplasia of the crypt and provided soy peptides, amino acids, and lactic acids as a direct nutritive recourse to intestine villi or regulated microbiota indirectly to re-establish the gut epithelium. MUC2 secreted by gut goblet cells provides nutrients to the native microbiota and cushions between the cavities of epithelial cells and is primarily affected by the changing of microbiota [27]. In the study, FSBM_B_ had upregulated expression of *MUC2* and was beneficial to native microbiota proliferation.

The tight junction is related to osmosis in intestinal epithelial cells. When intestinal osmosis is enhanced, more potential pathogens can pass through the epithelial cells and cause inflammation [28]. The factors that cause gut injury (ROS from chyme and cell metabolism, and nutritional diarrhea) and *NF-κB*, which inhibit *zonula occludens 1* (*ZO-1*) expression lead to impeded tight junction repair [29]. The allergen fragment in the SBM storage protein inhibits tight junction repair by the active *NF-κB* pathway [28]. Peng [30] showed that β-conglycinin is harmful to the maintenance of the tight junction. Soy peptides only protect from injury due to allergens, pathogens, or other signals, but could not restore the situation before damage [31]. Zhang [15] also proved that soy peptides could support intestinal epithelial repair in piglets by decreasing ROS and *NF-κB* expression. In this study, we did not induce inflammation by 4 4′-Diaminodiphenylsulfone or other pathogens. All three treatments upregulated *ZO-1*, *claudin-1*, and *occludin* expression, which improved and stabilized the tight junction barrier.

β-conglycinin from SBM can enhance innate inflammation-related mRNA levels, including those of *NF-κB* [30]. In the jejunum, FSBM_L_ has the highest mRNA-related level of *NF-κB* and upregulated *IL-6* from the CD4+ T cell and leads to tissue inflammation [32]. FSBM_L_ also upregulated the IL-1β and IL-10 levels. IL-1β was synthesized from macrophages by accepting pathogen-associated molecular patterns and secondary signals (ROS, crystals, or potassium efflux from cell damage), and upgrades the T cell *NF-κB* expression. The other cytokine, IL-10, from the Treg cell, can regulate the overexpression by IL-1β and cause innate inflammation [32,33]. The reason for this upgradation of innate inflammation by FSBM_L_ may be ascribed to minimal degradation of allergen protein and induction of the pro-inflammatory cytokine IL-6 by Lb [34].

On the other hand, the levels of IL-6 and IL-1β were enhanced in FSBM_B_. The degradation of glycinin and β-conglycinin fragments could possibly still stimulate the immune cells, but not enough to synthesize pro-inflammatory cytokines. The upregulation of *IFN-γ* and *IL-10* showed that soy peptides from FSBM_B_ could still promote self-healing and immune-regulated ability in intestinal epithelial cells. IFN-γ from T helper cell 1 could inhibit and clear the damaged cells [35]. Furthermore, the soy peptides could support appropriate expression in the dextran sodium sulfate (DSS)-damaged epithelial cells of piglets [5]. Among other treatments, unlike FSBM_B_, FSBM_B+L_ had minimal upregulation of IL-6. After allergen protein was hydrolyzed by protease or fermentation, the lactic acid bacteria could reduce the peptides into a smaller size and increase their bioactivity [36,37,38]. Likewise, Ren [31] also indicated that soy peptide contains a large percentage of Glu and Asp that can down-regulate *NF-κB*, which causes an innate inflammatory response, and help repair the gut injury. 

In serum, we did not detect a sufficient amount of IL-10 and IL-6 proteins. However, we detected the highest level of IL-1β in the SBM group, which is considered to stimulate other organs via β-conglycinin subunit fragments such as Gly m Bd 30K, Gly m Bd 60K, and Gly m Bd 28K [16,39]. FSBM_B_ had a high content of soy peptides that could pass through the intestine epithelium and enter the circulatory system to increase the stimulation of IL-1β in the FSBM group more than in FSBM_L_ and FSBM_B+L_, but was significantly decreased in SBM because of the elimination of the allergen. In Cheng’s group experiment [18], fermented SBM decreased the anti-nutrition factor while increasing < 6 kDa soy peptides contents. Additionally, 10% partial replacement FSBM of feed downregulated *IL-4* and *IL-10* in the spleen and IgG levels in serum due to degradation of anti-nutrition factors. However, they did not further describe the positive effect to immunity from their fermented soy peptides. FSBM_B_, which, degrades anti-nutritional factors and increases peptide content. However, our experiments have shown that fermentation strains with site fermentation conditions that strongly degrade allergic protein factors and producing soybean peptides cannot be fully equipped to regulate the function of intestinal immunity. We suggest that cooperation of functional strains may fix the peptides that improve the peptide bioactive activity, but we still need further investigation to support our hypothesis, such as separation and identification of specific fragments.

Although the degradation of soybean structural protein in FSBM_B_ and FSBM_B+L_ is higher than FSBM_L_, we still observed that FSBM_B_ and FSBM_B+L_ and its TCA-soluble protein stimulate the expression of *IL-1β*, *IL-6*, and *IL-10* to a certain extent in the jejunum. The residues of the decomposed soy peptides may partially stimulate the innate immune response, which will help animals accelerate the immune system’s activation when facing acute or chronic stress [40].

So far, the ability of soy peptides to repair or balance gut injury could be tested through in vitro study or with in vivo experiment [14,15]. In Zhu’s studies [41], they showed that FSBM soy peptides affect animals’ immune responses by measuring the TCA-soluble protein, and the expression of LC3B from the jejunum and ileum in the piglets correlated with soy peptides, which inhibit the ROS overexpression and cause innate immunity and gut injury. In this study, the FSBM_B+L_ and FSBM_B_ TCA-soluble proteins show positive traits regarding immunity of the jejunum and tight junction through molecular mechanisms.

## 5. Conclusions

The FSBM fermented by Bv or through a two-stage combination with Lb could increase the TCA-soluble protein and reduce the anti-nutrition factors (GOS, allergen protein) of the feed. Partial replacement of 6% of broiler diet by FSBM improved the intestinal traits, maintaining the expression of tight junction-related genes while stimulating the expression of inflammatory factor and MUC2 in the jejune. Compared to FSBM_L_, FSBM_B+L_ exhibited less *NF-κB* and *IL-6* related expression, indicating better intestinal repairability in animals facing potential environmental stress and maintaining good growth performance.

## Figures and Tables

**Table 1:** Chemical composition of SBM and two-stage FSBM products.

Nutrient	SBM	FSBM_L_	FSBM_B_	FSBM_B+L_	SEM	*p*-Value
Composition						
DM (%)	88.4 ^c^	93.2 ^a^	88.2 ^c^	89.9 ^b^	0.001	<0.001
CP (% DM)	43.0	45.1	50.8	48.1	1.72	0.104
*TCA*-soluble protein (% DM)	4.21 ^d^	7.42 ^c^	15.17 ^a^	10.29 ^b^	0.49	<0.001
Lactic acid Bacteria (Log CFU/g DM)	5.54 ^c^	8.82 ^a^	8.00 ^b^	8.97 ^a^	0.08	<0.001
Anti-nutritive factors						
Raffinose (% DM)	1.29 ^a^	0.11 ^b^	0.08 ^b^	0.0 3^b^	0.04	<0.001
Stachyose (% DM)	4.15 ^a^	1.19 ^b^	ND	ND	0.08	<0.001
Allergen protein (mg/g DM)	505 ^a^	226 ^b^	183 ^b^	198 ^b^	35.8	0.001
Trpsin inhibitor (mg/g DM)	17.5 ^a^	9.03 ^b^	0.43 ^d^	1.57 ^c^	0.16	<0.001

Each value represents the mean ± standard deviation (n = 3). ND: Not detected. SBM: Soybean meal; FSBM_L_: commercial control; FSBM_B_: SBM one-stage fermented by Bv; FSBM_B+L_: SBM two-stage fermented by Bv and Lb. ^a–d^ Means within a row with different letters differed significantly (*p <* 0.05).

**Table 2:** Composition and calculated analysis (% as fed) of the basal diet for broilers (1–35 days) ^1^.

Ingredients	Starter Diet (Day 1–21)				Finisher Diet (Day 22–35)			
	SBM	FSBM_L_	FSBM_B_	FSBM_B+L_	SBM	FSBM_L_	FSBM_B_	FSBM_B+L_
**Composition, %**								
Corn, yellow	52.99	53.27	54.18	53.49	57.15	57.39	58.45	57.61
Soybean meal (CP-44%)	34.0	28.0	28.0	28.0	28.0	22.0	22.0	22.0
Fermented soybean meal	-	6.0	6.0	6.0	-	6.0	6.0	6.0
Full fat soybean meal	3.00	2.99	2.00	2.47	4.15	4.18	3.04	3.65
Soybean oil	3.16	2.89	2.97	3.20	4.13	3.86	3.93	4.17
Fish meal (CP-65%)	3.00	3.00	3.00	3.00	3.00	3.00	3.00	3.00
Monocalcium phosphate	1.32	1.32	1.32	1.32	1.25	1.25	1.25	1.25
Calcium carbonate	1.36	1.36	1.36	1.36	1.28	1.28	1.28	1.28
NaCl	0.34	0.34	0.34	0.34	0.34	0.34	0.34	0.34
_DL_-Methionine	0.35	0.35	0.35	0.35	0.27	0.27	0.27	0.27
_L_-Lysine HCl	0.20	0.20	0.20	0.20	0.16	0.16	0.16	0.16
Choline-Cl	0.08	0.08	0.08	0.08	0.08	0.08	0.08	0.08
Vitamin premix ^2^	0.10	0.10	0.10	0.10	0.10	0.10	0.10	0.10
Mineral premix ^3^	0.10	0.10	0.10	0.10	0.10	0.10	0.10	0.10
Total	100.0	100.0	100.0	100.0	100.0	100.0	100.0	100.0
Calculated nutrient levels								
Crude protein, %	23.0	23.0	23.0	23.0	21.0	21.0	21.0	21.0
Crude fat, %	6.6	6.4	6.3	6.6	7.8	7.7	7.6	7.8
ME, kcal/kg	3050.0	3050.0	3050.0	3050.0	3175.0	3175.0	3175.0	3175.0
Calcium, %	0.96	0.96	0.96	0.96	0.90	0.91	0.91	0.91
Total phosphorus, %	0.70	0.71	0.71	0.71	0.66	0.68	0.67	0.67
Available phosphorus, %	0.48	0.47	0.46	0.46	0.46	0.44	0.44	0.44
Methionine,+Cysteine, %	1.08	1.08	1.07	1.07	0.95	0.95	0.94	0.95

^1^ SBM: Soybean meal; FSBM_L_: commercial control; FSBM_B_: SBM one-stage fermented by Bv; FSBM_B+L_: SBM two-stage fermented by Bv and Lb. ^2^ Vitamins (premix content per kg diet): Vit. A, 15,000 IU; Vit. D3, 3000 IU; Vit. E, 30 mg; Vit. K3, 4 mg; thiamine, 3 mg; riboflavin, 8 mg; pyridoxine, 5 mg; Vit. B12, 25 μg; Ca-pantothenate, 19 mg; niacin, 50 mg; folic acid, 1.5 mg; and biotin, 60 μg. ^3^ Minerals (premix content per kg diet): Co (CoCO_3_), 0.255 mg; Cu (CuSO_4_·5H_2_O), 10.8 mg; Fe (FeSO_4_·H_2_O), 90 mg; Mn (MnSO_4_·H_2_O), 90 mg; Zn (ZnO), 68.4 mg; Se (Na_2_SeO_3_), 0.18 mg.

**Table 3:** The primer sequence of each gene according to Genbank or other research.

Gene Name ^1^	Primer Sequence	Genbank No.
ß-actin	F: 5′-CTGGCACCTAGCACAATGAA-3′ R: 5′-ACATCTGCTGGAAGGTGGAC-3′	X00182.1
NFκB	F: 5′-CCAGGTTGCCATCGTGTTCC-3′ R: 5′-GCGTGCGTTTGCGCTTCT-3′	D13719.1
IFN-γ	F: 5′-CTCCCGATGAACGACTTGAG-3′ R: 5′-CTGAGACTGGCTCCTTTTCC-3′	Y07922
IL-1ß	F: 5′-GCTCTACATGTCGTGTGTGATGAG-3′ R: 5′-TGTCGATGTCCCGCATGA-3′	NM_204524
IL-6	F: 5′-AGGACGAGATGTGCAAGAAGTTC-3′ R: 5′-TTGGGCAGGTTGAGGTTGTT-3′	NM_204628
IL-10	F: 5′-TTCAGCTTGGATGTGTGAGC-3′ R: 5′-TGTCAGTTCTGCATGCTTCC-3′	XM_025143715.1
Claudin-1	F: 5′-GGAGGATGACCAGGTGAAGA-3′ R: 5′-TCTGGTGTTAACGGGTGTGA-3′	NM_001013611.2
MUC-2	F: 5′-GCTACAGGATCTGCCTTTGC-3′ R: 5′-AATGGGCCCTCTGAGTTTTT-3′	NM_001318434.1
Occludin	F: 5′-GTCTGTGGGTTCCTCATCGT-3′ R: 5′-GTTCTTCACCCACTCCTCCA-3′	NM_205128.1
ZO-1	F: 5′-AGGTGAAGTGTTTCGGGTTG-3′ R: 5′-CCTCCTGCTGTCTTTGGAAG-3′	XM_015278975.1

^1^ NFκB: Nuclear factor kappa B p 65; IFN-γ: Interferon-γ; IL-1ß: Interleukin-1ß; IL-6: Interleukin-6; IL-10: Interleukin-10; MUC-2: Mucin2; ZO-1: Zonula occludens 1.

**Table 4:** Effect of two-stage FSBM on intestinal morphology of 35 day-old broilers ^1^.

Items	Experimental Diet					
	SBM	FSBM_L_	FSBM_B_	FSBM_B+L_	SEM	*p*-Value
Jejunum						
Villus height (μm)	1181 ^b^	1326 ^a^	1293 ^a^	1312 ^a^	26.2	<0.001
Crypt depth (μm)	210 ^a^	206 ^a^	175 ^b^	196 ^a^	5.38	<0.001
Villus height/crypt depth	5.62 ^c^	6.44 ^b^	7.39 ^a^	6.69 ^b^	0.18	<0.001
Ileum						
Villus height (μm)	955	971	965	985	17.5	0.676
Crypt depth (μm)	202	204	204	193	5.79	0.461
Villus height/crypt depth	4.73	4.76	4.73	5.10	0.15	0.125

^1^ Each value represents the mean ± standard deviation (n = 6). SBM: Soybean meal; FSBM_L_: commercial control; FSBM_B_: SBM one-stage fermented by Bv; FSBM_B+L_: SBM two-stage fermented by Bv and Lb. ^a–c^ Means within a row with different letters differed significantly (*p* < 0.05).

**Table 5:** Effect of two-stage FSBM on serum and jejunum’s immunity of 35 day-old broilers ^1^.

Items	Experimental Diet					
	SBM	FSBM_L_	FSBM_B_	FSBM_B+L_	SEM	*p*-Value
Jejunum						
IL-1β (pg/mg protein)	10.2 ^b^	16.9 ^a^	11.7 ^b^	12.5 ^b^	1.15	0.016
IL-10 (pg/mg protein)	134 ^b^	208 ^a^	130 ^b^	135 ^b^	4.44	<0.001
IL-6 (pg/mg protein)	655 ^b^	769 ^a^	510 ^c^	500 ^c^	36.3	0.002
Serum						
IL-1β (pg/mL)	90.1 ^a^	43.1 ^c^	70.9 ^ab^	51.5 ^bc^	7.95	0.018

^1^ Each value represents the mean ± standard deviation (n = 5). SBM: Soybean meal; FSBM_L_: commercial control; FSBM_B_: SBM one-stage fermented by Bv; FSBM_B+L_: SBM two-stage fermented by Bv and Lb. ^a–c^ Means within a row with different letters differed significantly (*p* < 0.05).

**Table 6:** Evaluation of the economic benefit of two-stage FSBM supplemented in diet.

Item	Experimental Diets			
	SBM	FSBM_L_	FSBM_B_	FSBM_B+L_
Feed cost, TWD/bird				
1–35 days	46.6	49.0	49. 6	49.3
Meat income, TWD/bird				
1–35 days	108.6	108.9	106.8	112.6
Income over feed cost, TWD/bird				
1–35 days	61.9	59.9	57.2	63.3

Feed cost, based on the costs (TWD/kg) of the ingredients, as follows: corn meal, 10.1; soybean meal, 15.0; fermented soybean meal, 23.5; full fat soybean meal, 18.2; soybean oil, 40.0; fish meal, 42.0; monocalcium phosphate, 14.5; calcium carbonate, 1.70; salt (NaCl), 4.5; DL-Methionine, 110.0; L-Lysine-HCl, 58.00; choline chloride, 50% 46; vitamin premix, 210.0; and mineral premix, 44.00. The fees for the processing of basal rations per kg were 14.54 for the grain mixture of the control group, 14.97 for the FSBM_L_ group, 14.92 for the FSBM_B_ group, and 15.02 for the FSBM_B+L_ group, respectively, over 1–21 days. The fees for the processing of basal rations per kg were 14.54 for the grain mixture of the control group, 14.97 for the FSBM_L_ group, 14.90 for the FSBM_B_ group, and 15.02 for the FSBM_B+L_ group, respectively, over 22–35 days.

## Data Availability

The data presented in this study are available on request from the corresponding author.

## Abbreviations

FSBM	Fermented soybean meal
Bv	*Bacillus velezensis*
Lb	Lactobacillus brevis ATCC 367
FSBM_B+L_	Two-stage fermented soybean meal by *Bacillus velezensis* and *Lactobacillus brevis* ATCC 367
SBM	Soybean meal
GOS	Galacto-oligosaccharide
FSBM_B_	One-stage fermented soybean meal by *Bacillus velezensis*
FSBM_L_	Commercial fermented soybean meal products fermented by *lactobacillus spp.*
CLDN-1	Claudin-1
ZO-1	Zonula occludens 1
NFκB	Nuclear factor kappa B p 65
IL-6	Interleukin-6
MUC2	Mucin2
ACE-inhibitory	Angiotensin-converting-enzyme inhibitory
Th1	T Helper 1 Cells
IL-12	Interleukin-
TNF-α	Tumor Necrosis Factor-α
IFN-γ	Interferon-γ
TGF-β	Transforming Growth Factor Beta
IL-17	Interleukin-
IL-10	Interleukin-
PepT-1	Peptide transporter 1
SEM	Standard error of mean
TCA- soluble protein	Trichloroethanoic acid- soluble protein
BW	Body weight
FI	Feed intake
BWG	Body weight gain
FCR	Feed conversion rate
IL-1ß	Interleukin-1ß
ROS	Reactive oxygen species
